# Educational Review: Error traps in anesthesia for pediatric liver transplantation

**DOI:** 10.1111/pan.14565

**Published:** 2022-10-12

**Authors:** Heather A. Ballard, Elin Jones, Marcella M. Malavazzi Clemente, Daniela Damian, Pete G. Kovatsis

**Affiliations:** ^1^ Department of Pediatric Anesthesiology, Ann & Robert H. Lurie Children's Hospital of Chicago Northwestern University Feinberg School of Medicine Chicago IL USA; ^2^ Department of Anaesthesia Birmingham Children's Hospital Birmingham UK; ^3^ Serviços Médicos de Anestesia SMA Hospital Sírio‐Libanês São Paulo Brazil; ^4^ Department of Anesthesiology UPMC Children's Hospital of Pittsburgh and University of Pittsburgh School of Medicine Pittsburgh Pennsylvania USA; ^5^ Department of Anesthesiology, Critical Care and Pain Medicine Boston Children's Hospital and Department of Anaesthesia, Harvard Medical School Boston Massachusetts USA

**Keywords:** anesthesia, cirrhosis, coagulopathy, end stage disease, liver, Liver, liver transplantation, pediatrics

## Abstract

Anesthetic and surgical techniques for the liver transplantation have progressed considerably over the past sixty years; however, this procedure is still fraught with substantial morbidity. To increase the safety culture associated with the liver transplantation, we detail nine error traps associated with anesthesia for pediatric liver transplantation. These potential pitfalls are divided into the operative phases: pre‐operative preparation (*Failure to have a dedicated anesthesia team for pediatric liver transplantation*); pre‐anhepatic (*Failure to prepare for massive blood loss, Failure to monitor for coagulation abnormalities*); anhepatic including reperfusion (*Failure to prepare for clamping of the inferior vena cava*, *Failure to recognize metabolic changes, Failure to maintain homeostasis for reperfusion, Failure to prepare for Post‐reperfusion syndrome*); and post‐anhepatic (*Failure to optimize liver perfusion, Failure to maintain hemostatic balance*). By offering practical advice on the preparation and treatment of these error traps, we aim to better prepare anesthesiologists to take care of pediatric patients undergoing the liver transplantation.

## 1 INTRODUCTION

Nearly 60 years ago, the first liver transplant was attempted on a pediatric patient and within a few years, pediatric patients showed that extended survival after transplant was possible.^1^ Despite decades of advancements in knowledge and skills improving long‐term survival rates, the perioperative course continues to have a high potential for complications and iatrogenic adverse events.^2^ There are varied indications for proceeding with pediatric liver transplantation which has been delineated.^3^ The underlying cause necessitating the transplant determines what to anticipate with the procedure. These cases are prone to major fluid shifts, massive blood loss, rapid hemodynamic swings necessitating fluid resuscitation, blood products, and vasopressor support, while facing acute on chronic coagulation abnormalities, and imbalances in electrolyte and metabolic parameters. Patients with cirrhosis and resultant portal hypertension are at the most risk for these issues.

Every organ system may be affected by end‐stage liver disease, potentially resulting in encephalopathy, cerebral edema, hepatorenal syndrome, hepatopulmonary syndrome and pulmonary hypertension leading to increased peritransplant morbidity and mortality. Pediatric anesthesiologists deal with uncommon diseases of the liver and their associated comorbidities, requiring knowledge and expertise of the respective anesthetic considerations. The aim of this manuscript is to introduce the anesthesiologist to some of the numerous potential challenges and error traps that may occur during the pediatric liver transplant.^4^ The manuscript organizes the error traps into Preparation and the main stages of a liver transplant: Pre‐anhepatic, Anhepatic including Reperfusion and Post‐anhepatic (Post‐reperfusion).

## 2 PREPARATION

### 2.1 Error Trap 1.1: Failure to have a dedicated team for pediatric liver transplantation

Pediatric liver transplantation requires a multidisciplinary team to care for complex patients safely and successfully during this highly specialized and challenging procedure.^5^ A dedicated team that included anesthesiologists specializing in the perioperative care of liver transplantation was associated with improved patient outcomes at both adult and pediatric transplant centers.^6^, ^7^ The insights gained by a designated anesthesia team led to improved patient blood management (PBM) and decreased ventilator days from increased early extubation. The importance of having a consistent team of surgeons, nurses, and anesthesiologists cannot be underestimated. This familiarity allows for seamless, structured communications, and anticipation of events, which is paramount in the rapidly changing and complex environment of liver transplantation. Clear, constant, and unambiguous communication and case debriefings between team members require planning and practice within a culture of safety.

Most pediatric liver transplant centers perform less than forty cases per year. A dedicated team may play an even more important role at low‐volume institutions by allowing a core group of anesthesiologists to gain greater experience and expertise in the procedure and the varied clinical presentations. Such expertise should ensure that patients presenting for liver transplantation undergo a thorough pre‐anesthetic assessment to optimize the patient's medical status and prepare for potential pitfalls, including changes in cardiopulmonary status, metabolic derangements, challenges with vascular access, coagulopathy management, and administration of blood products. Transplant anesthesiologists complement the knowledge and experience of the surgeons and hepatologists during patient selection and preoperative planning to help to identify preoperative issues and optimize patient preparation. By recognizing and addressing these issues prior to surgery, the anesthesiologist also obtains critical information improving the anticipation of potential problems and the avoidance or mitigation of intraoperative events.

## 3 PRE‐ANHEPATIC PHASE

The Pre‐anhepatic phase starts with induction and ends with the occlusion of blood supply to the native liver: clamping the hepatic artery, portal vein, and inferior vena cava.

### 3.1 Error Trap 2.1: Failure to prepare for massive blood loss

Massive blood loss is a common challenge of pediatric liver transplantation. Children who have growth restriction, high‐pediatric end‐liver disease (PELD) scores, thrombocytopenia, and multiple previous abdominal procedures are at an increased risk.^8^, ^9^ Liver transplant recipients must have an adequate supply of blood products readily available in case of massive blood loss. The risk of massive transfusion necessitates appropriate invasive monitors to detect rapid hemodynamic changes and appropriate intravenous access to allow for rapid volume resuscitation. As blood products are a limited resource, discuss cell saver use with the surgical team preoperatively. History of cancer and active infection (cholangitis) are relative contraindications to the use of blood salvage techniques.^10^ Additionally, cell saver equipment can wash older packed red blood cells to lower the risk of hyperkalemia. It is critical to remember that cell‐saver blood has no plasma components, so one must be vigilant to transfuse plasma products in a rapid blood loss situation or coagulopathy will worsen.

Due to the rapidly changing physiological states encountered in liver transplantation, monitoring and testing devices that deliver moment‐to‐moment data are necessary. Transesophageal echocardiography (TEE) is rarely used in pediatric liver transplantation as compared to adult transplantation. However, echocardiography may be helpful to diagnose unexplained hemodynamic instability or carefully titrate blood products and fluids in patients with underlying cardiac disease. Pediatric cardiology consultation may be warranted to employ TEE if a practitioner is not certified for such use. A novel, hands‐free, transthoracic biplane echocardiography device (Terason uSmart 3200 T Ultrasound System; Burlington, MA) may supplant TEE in pediatric cases as it allows continuous, rapid assessment of volume status and cardiac function while avoiding the potential pitfalls and complications associated with TEE.^11^ Use of dynamic indexes such as pulse pressure variation and pulse variability index may aid in volume administration. Finally, the use of viscoelastic testing such as thromboelastography(TEG, Haemonetic, Boston, MA, USA), thromboelastometry (ROTEM, Instrumentation Laboratories, Bedford, MA, USA) can help guide the administration of blood products to treat coagulopathy (fresh frozen plasma [FFP] versus cryoprecipitate versus platelets).

Vascular access requirements:

1. Central venous access according to the patient weight (see Table 1)
2. Arterial access. Institutions vary in whether to place one or two arterial lines. The advantage of placing two arterial lines is that during periods of significant volume and hemodynamic swings, one line continuously monitors the patient's blood pressure while the other is for blood draws. (see Table 2)
3. Obtain peripheral intravenous (PIV) access above the diaphragm due to the potential of inferior vena cava clamping. Place at least two wide bore, PIV catheters with one typically connected to a high‐pressure rapid infusion system.
4. If massive blood administration is anticipated, consider placing an introducer either peripherally if feasible or centrally (preferably on the right jugular vein due to straighter trajectory). See Table 1 for size recommendations based on age.
5. Using ultrasound is recommended for arterial and central access, and difficult peripheral intravenous access [PIV] to reduce the number of attempts, time to successful placement, and increase safety in those children with pre‐existing coagulation issues.^12^ As ultrasound assesses the size of the vein, it facilitates the placement of larger bore catheters.
6. Discussion with the surgeons of their plan for venous and arterial grafting will facilitate decision‐making as to whether venous and arterial access below the diaphragm will be acceptable.
7. Access obtained for veno–veno bypass does not count toward resuscitation access.

**TABLE 1:** Recommendations for peripheral and central venous access

Age	Weight (kg)	Peripheral venous access^a^	Central venous access^b^			Introducer^c^	
		Gauge size	French size	Length (cm)	Lumens	Length (cm)
0–28 days	2–5	24; 22	4	5	2	5	5
1–12 months	<10	22; 20	4	8	2	5	7.5
1–8 years	10–30	20; 18; 16	5	12	3	6	7.5
8–14 years	30–50	18; 16; RIC^d^	5–7	15	3	6	11
Teenager	>50	16; 14; RIC^d^	7	15–20	3	7–8	11

^a^ Preferably with ultrasound guidance, begin distal and move proximal to the cephalic or basilic veins as indicated; consider using longer than standard length peripheral catheters.

^b^ Preferably right internal jugular.

^c^ If massive transfusion anticipated, consider placing an introducer in one of the jugular veins, preferable on the right.

^d^ RIC: Rapid infusing catheter ‐ 7F/ 5 cm.

**TABLE 2:** Recommendations for arterial access

Weight (kg)	Peripheral artery^a^ ^,^ ^b^	Femoral artery	
	Gauge size	French size	Length (cm)
<3	24	2.5	5
3–10	22	2.5	5
10–30	22	3	8
30–50	20	3	8
>50	20	4	12

^a^ If arterial caliber small, consider smaller gauge and longer than standard catheters.

^b^ Upper extremity arterial access is often preferred due to potential cross clamping of aorta, but other sites (ulnar, posterior tibial, and dorsalis pedis) have also been utilized.

### 3.2 Error Trap 2.2: Failure to monitor for coagulation abnormalities

Advancements in surgery, anesthesia, and critical care has led to a dramatic reduction in transfusion requirements in the pediatric liver transplant recipients.^13^ This includes an evolving understanding of the complex balance between pro‐ and anti‐coagulation pathways in liver failure. Prevention of thrombotic complications is as important as the management of hemorrhage.^14^ Overcorrection of coagulopathy can occur when the severity of coagulopathy is over‐estimated or with rapid transfusion of coagulation products, particularly during a period of substantial blood loss. This can be the result of insufficient frequency of testing, reliance on traditional indices such as INR, PT, and PTT, and inadequate usage of viscoelastic testing. These tests take a significant amount of time for results and the clinical picture may change during this time. Viscoelastic hemostatic assays such as TEG, ROTEM, and most recently the sonorheometry‐based Quantra Hemostasis Analyzer (HemoSonics, Charlottesville, VA, USA), are effective in guiding perioperative blood management by reducing both the transfusion of allogeneic blood products and costs.^13^, ^15^

As standardized protocols for transfusion in the pediatric liver transplant are not established, local clinical practice continues to prevail. Use fresh frozen plasma, cryoprecipitate, and platelets judiciously due to concerns for hepatic artery and portal vein thrombosis. Changes in blood product utilization reflected global trends and coincided with the introduction of locally formulated massive transfusion protocols that directed the early use of blood products to correct coagulopathy based on laboratory testing.^16^ Fibrinogen depletion is common in children with fulminant hepatic failure. If excessive surgical bleeding occurs, monitor fibrinogen levels and correct with cryoprecipitate or administration of purified fibrinogen concentrate. Although current patient blood management guidelines support the administration of fibrinogen if below 150–200 mg/dL or maximum clot firmness is low in viscoelastic testing,^17^ anesthesiologists must correlate laboratory findings with clinical signs of coagulopathy to determine optimal blood product administration given the changing balance in coagulation throughout the transplant. Platelet therapy is reserved for treating platelet counts less than 25–50 000 cells/mL unless there is excessive surgical bleeding.

No clear recommendation can be made regarding the indication for prothrombin complex concentrate (PCC) or FXIII, and the use of rFVIIa and desmopressin in the absence of hemophilia are not recommended.^18^ Intraoperative prophylactic administration of antifibrinolytics in pediatric liver transplant is not advocated due to concerns of thrombosis. Clinical outcome studies are needed for the development of pediatric‐specific algorithms to guide clinical management of bleeding disorders and transfusion therapy during the pediatric liver transplantation.^16^

## 4 ANHEPATIC

The anhepatic phase starts with the occlusion of blood flow to the diseased liver and ends with reperfusion of the new graft. Typically, the new liver is perfused with the unclamping of the inferior vena cava (outflow) and portal vein (inflow). The hepatic artery anastomosis is performed during the post‐anhepatic phase to reduce the graft's cold ischemic time.

### 4.1 Error Trap 3.1: Failure to prepare for clamping of the inferior vena cave (IVC)

To facilitate hepatectomy and surgical vascular anastomoses, the IVC is clamped. The conventional method is to place vascular clamps on the infrahepatic and suprahepatic IVC. A popular alternative is the piggyback technique, where the IVC is partially or side‐clamped to allow some blood flow during clamping which may result in less hemodynamic instability.^19^

Clamping the IVC can lead to severe hypotension with loss of up to 50% of the preload to the heart.^19^ Patients without portal hypertension (i.e., metabolic disease, toxins, infectious hepatitis) may have an exaggerated response to clamping as they lack variceal collaterals that bypass the normal liver drainage in the IVC secondary to portal hypertension. The goals are a low/normal central venous pressure (≤7‐10 mmHg) and hemoglobin (8‐10 g/dL). Hypotension is preferentially treated with vasopressors instead of IV fluids to avoid the risk of volume overload, right heart failure, and graft congestion during reperfusion.^20^ Judicious use of intravenous fluids is required as replacing significant volume because of the temporary decrease of venous return from the lower body from the IVC clamp may result in hypervolemia soon after reperfusion.

### 4.2 Error Trap 3.2: Failure to recognize metabolic changes

The anhepatic period marks the complete lack of liver function; therefore, any metabolic issues during the pre‐anhepatic phase are amplified during this phase. Electrolyte abnormalities are common and exacerbated by rapid blood transfusion. Acidosis results from the loss of the liver's capacity to metabolize lactate, along with the need for acid‐containing blood transfusions.^21^ The decreased pH level and potassium load from blood transfusions increase the patient's risk for hyperkalemia; however, hyperkalemia is less common in the pediatric liver transplantation.^22^ The inability to metabolize citrate, a blood preservative, leads to calcium binding and hypocalcemia causing loss of vascular tone and decreased heart contractility.^23^ Hypoglycemia is also common during the anhepatic stage due to a lack of gluconeogenesis and loss of glycogen stores combined with an inability to degrade insulin.^21^ In addition, the patient is at risk for hypothermia, typically losing an additional 1–2°C as the cold graft enters the abdomen^23^ (Table 3).

**TABLE 3:** Treatment of anhepatic phase derangements

Anhepatic abnormality	Treatment
Hypotension	Keep CVP at low normal (7–10 mmHg) Use vasopressors to augment blood pressure and avoid over transfusion/fluid administration
Hypothermia	Use fluid warmers, warming cap, convection air warming devices, and if necessary, warm the room
Metabolic Acidosis	Treatment with sodium bicarbonate (1 mEq/kg) to maintain near normal pH
Hypocalcemia	Supplementation with calcium chloride (10 mg/kg, central access) or calcium gluconate (30 mg/kg, central access or peripheral) to keep ionized calcium >1.2 mmol/L
Hyperkalemia	Aim for low normal potassium levels (3–4 mEq/L) in preparation for reperfusion Consider using newer or washed blood products Treatment with hyperventilation (PCO_2_‐30 mmHg), albuterol (4 puffs), insulin (0.1 units/kg) with dextrose (0.25–0.5 g/kg), furosemide (0.5–1 mg/kg), sodium bicarbonate, and calcium chloride/gluconate, renal replacement therapy if available
Hypoglycemia	Frequent blood sampling of glucose levels Supplementation with a continuous glucose infusion and bolus, if needed, to keep blood sugar >80 mg/dL

### 4.3 Error Trap 3.3: Failure to maintain homeostasis for reperfusion (overtreat with expectation)

The anesthesiologist must be vigilant in treating metabolic derangements during this phase, with the expectation that reperfusion will bring a large bolus of cold, potassium‐containing, acidotic fluid from the ischemic graft and the stagnant blood distal to the caval clamp.^24^ At some centers, the surgeon will flush the donor graft with a balanced crystalloid solution prior to implantation to reduce these hemodynamic perturbations. The surgeon should communicate this maneuver to the entire team to help facilitate preparation for reperfusion. The treatment of metabolic derangements during the anhepatic phase is detailed in Table 3. Frequent blood sampling every 15–20 minutes helps to determine how to optimize the patient for reperfusion. Calcium and bicarbonate supplementation are often required to counteract the increased citrate and acid levels from the transfusion of blood products. We recommend keeping potassium at low/normal levels (3–4 mEq/L) in preparation for reperfusion. (Table 3).

### 4.4 Error Trap 3.4: Failure to prepare for post‐reperfusion syndrome

Post‐reperfusion syndrome (PRS) is a serious intraoperative complication that occurs following the release of the portal vein and caval clamps. Initially defined as an isolated fall in arterial blood pressure of more than 30%, PRS is a complex physiologic process including mitochondrial de‐energization, metabolic acidosis, adenosine triphosphate depletion, Kupffer cell activation, calcium overload, oxidative stress, and the upregulation of pro‐inflammatory cytokine signal transduction.^25^ Various donor, recipient, surgical, and therapeutic factors contribute to PRS (see Table 4). PRS increases perioperative morbidity and mortality, blood product transfusions, postoperative kidney injury, and ICU and hospital length of stay. PRS contributes to postoperative graft dysfunction, especially affecting bile ducts that are highly susceptible to oxygen deprivation and reperfusion injury and can start a cascade that leads to multiple organ dysfunction.

**TABLE 4:** Factors contributing to Post‐reperfusion syndrome (PRS)

Donor factors^25^	Recipient factors^26^	Surgical and therapeutic factors
Ischemia–reperfusion injury	Elevated Model for End‐Stage Liver disease (MELD) score	Surgical technique: reduced with piggy‐back technique together with a blood flush
Hypertension	Elevated creatinine	The cytokine response^a^
Increased donor age	Elevated potassium	Blocking activation of the immune system^a^
Hepatic steatosis	Low calcium	Increased cold ischemic time (may be mitigated by ex vivo perfusion)^b^
Nature of organ recovery^30^ ^,^ ^a^	Low hemoglobin	Lack of N‐Acetyl cysteine and increased prostaglandins and TNF‐alpha^b^
Extended criteria grafts	Size mismatch between donor and recipient with increases seen in patients transplanted with large organs for their size	

^a^ For deceased donors, increased travel distance, omission of pre‐mortem heparin, and longer asystole to cross‐clamp time may increase PRS.

^b^ Ongoing hypothesis under research.

During the first few minutes after graft reperfusion, significant hemodynamic changes can occur including a sudden decrease in blood pressure, heart rate, systemic vascular resistance, and changes in end‐tidal CO2.^24^ Acidotic and hyperkalemic sequestered blood from the portal and lower body venous system suddenly returns to the heart. From this abrupt systemic inflow of blood with metabolic and electrolyte derangements, the heart will be overloaded and its function depressed alongside systemic vasodilation and pulmonary vasoconstriction. To decrease the risk of PRS, optimize vascular volume and aggressively treat electrolyte and metabolic abnormalities, including additional intravenous calcium to stabilize the cardiac membrane before graft reperfusion.

Increased bleeding can also occur from the raw edge of a split donor graft, surgical complications, and coagulopathy worsened by ischemic by‐products, inflammatory mediators, and tissue plasminogen activators. Therefore, implement adequate volume resuscitation as clinically indicated with concurrent inotropes, vasopressors, and blood products for circulatory and coagulation support. Change FiO_2_ to 1.0 to increase oxygen stores and decrease volatile agents to minimize vasodilatation. Evidence surrounding strategies to correct the metabolic acidosis is inconclusive. While mild acidosis may be protective and self‐correct with reperfusion and subsequent function of the graft, moderate to severe acidosis should be treated. Most anesthesiologists will treat more aggressively prior to reperfusion as the effects of reperfusion: worsening acidosis, hyperkalemia, and impaired cardiac contractility may be attenuated with treatment.

## 5 POST‐ANHEPATIC

Reperfusion of the liver starts the post‐anhepatic phase, which consists of hepatic arterial anastomosis, biliary‐enteric anastomosis, and ends with abdominal closure. The multifaceted goals during this phase include optimizing liver perfusion and obtaining hemostatic balance while avoiding fluid overload.

### 5.1 Error Trap 4.1: Failure to optimize liver perfusion

Intravenous fluid therapy during postanhepatic phase is tailored for the maintenance of cardiac output and intravascular fluid volume while minimizing interstitial edema and the risk of thrombosis. Surgeons can directly observe the flow through the inferior vena cava and portal vein to provide feedback on perfusion and intravascular volume. Fluid overload may impair graft function and cause intestinal edema. Conversely, hypovolemia results in hypoperfusion to the new graft, which increases the risk of thrombosis and graft injury. Closure of the abdominal cavity under tension may also worsen graft perfusion and can lead to abdominal compartment syndrome, resulting in graft, and potentially multiple organ failure.

### 5.2 Error Trap 4.2: Failure to maintain hemostatic balance

Compared with adults, children are more hypercoagulable after liver transplant which may be due to decreases in proteins C and S, plasminogen, and anti‐thrombin III or elevated levels of factor VIII.^26^, ^27^ Hepatic artery thrombosis (HAT) occurs in up to 18% of pediatric recipients, which is as much as four times more frequent than in adult transplant patients, while PVT occurs in 5%–10% of recipients.^18^ Intraoperative HAT and early PVT are factors independently associated with decreased 90‐day patient survival.^28^

Conditions associated with an increased risk of vascular complications include presurgical portal vein thrombosis, cholestatic recipient diseases, Budd–Chiari disease, hypoplastic portal vein, living‐donor liver transplant, and split liver grafts, complex vessel reconstruction, intraoperative portal or hepatic artery thrombectomy, and decreased intraoperative portal or arterial blood flow. Patients with increased thrombotic risk may require perioperative thromboprophylaxis, despite being hypocoagulable.^14^ Throughout the procedure, the anesthesiologist must be aware of the patient's precarious coagulation status and continually rebalance the risk of bleeding versus the risk of clotting.

## 6 THE FOLLOWING PARAMETERS ARE PRACTICAL GOALS TO DEAL WITH THE VARIABLES DESCRIBED ABOVE

In order to preserve perfusion to the liver graft, maintain systemic mean arterial pressure in the range of 50–70 mmHg for infants, 60–80 mmHg for small children, or 70–90 mmHg for older children and adolescents.^20^ Higher blood viscosity associated with higher hematocrit levels may predispose to thrombosis, such that lower hemoglobin levels of 8–10 gm/dL are targeted.^23^ CVP is critical data in the post‐anhepatic phase as a measure of the complex interplay between venous return and cardiovascular impedance. Though CVP has been used to monitor intravascular volume, CVP readings can be misleading because of changes in intra‐abdominal pressure.^21^ Titrating fluid support is tenuous in this phase as systemic vascular resistance is low. Sufficient intravascular volume is needed to support adequate perfusion of the new graft, yet over‐transfusion of intravenous fluids can lead to graft congestion. CVP must be optimized to ensure adequate hepatic venous drainage and prevent graft congestion.

Volume replacement using an isotonic crystalloid solution compatible with blood is preferred. Some anesthesiologists prefer using albumin to keep fluids intravascularly; diluting 25% albumin with an isotonic fluid avoids the acidosis and hypernatremia associated with the administration of large volumes of normal saline when using 5% albumin. If fluid requirements remain high during this phase, communication with the surgical team is required to rule out obstruction of the liver and mesenteric vascular structures. Norepinephrine, epinephrine, dopamine, and phenylephrine are the vasoactive drugs most commonly used during this phase, individually or combined. Vasopressin is added if vasoplegia persists despite the administration of multiple vasopressors. Methylene blue dye has been used successfully to treat persistent vasoplegia.^29^

At the completion of the procedure, ensure adequate oxygenation and ventilation during abdominal closure while monitoring for continued bleeding and hemodynamic instability. Early extubation in the operating room immediately following liver transplantation is employed in selected subpopulations of pediatric patients, but it is unclear whether such extubation is a major factor affecting the length of PICU and hospital stays for the pediatric liver transplant patients.^30^

## 7 CONCLUSION

The pediatric liver transplantation is fraught with a high potential of serious adverse events and iatrogenic errors. A core group of pediatric anesthesiologists that specialize in transplantation is critical to success and reducing iatrogenic complications through expert team dynamics, structured communications, and an in‐depth understanding of the underlying pathophysiology that occurs during these complex operations, which leads to the anticipation of events with a focus on frequent and timely testing.

## Data Availability

Data sharing not applicable to this article as no datasets were generated or analyzed during the current study.
